# Pumilio Regulates Sleep Homeostasis in Response to Chronic Sleep Deprivation in *Drosophila melanogaster*

**DOI:** 10.3389/fnins.2020.00319

**Published:** 2020-04-17

**Authors:** Luis A. De Jesús-Olmo, Norma Rodríguez, Marcelo Francia, Jonathan Alemán-Rios, Carlos J. Pacheco-Agosto, Joselyn Ortega-Torres, Richard Nieves, Nicolás Fuenzalida-Uribe, Alfredo Ghezzi, José L. Agosto

**Affiliations:** Department of Biology, University of Puerto Rico, Rio Piedras, Puerto Rico

**Keywords:** sleep homeostasis, neuronal homeostasis, Pumilio, pum, *Drosophila*, chronic sleep deprivation, synaptic proteins, neuronal excitability

## Abstract

Recent studies have identified the *Drosophila* brain circuits involved in the sleep/wake switch and have pointed to the modulation of neuronal excitability as one of the underlying mechanisms triggering sleep need. In this study we aimed to explore the link between the homeostatic regulation of neuronal excitability and sleep behavior in the circadian circuit. For this purpose, we selected Pumilio (Pum), whose main function is to repress protein translation and has been linked to modulation of neuronal excitability during chronic patterns of altered neuronal activity. Here we explore the effects of Pum on sleep homeostasis in *Drosophila melanogaster*, which shares most of the major features of mammalian sleep homeostasis. Our evidence indicates that Pum is necessary for sleep rebound and that its effect is more pronounced during chronic sleep deprivation (84 h) than acute deprivation (12 h). Knockdown of *pum*, results in a reduction of sleep rebound during acute sleep deprivation and the complete abolishment of sleep rebound during chronic sleep deprivation. Based on these findings, we propose that Pum is a critical regulator of sleep homeostasis through neural adaptations triggered during sleep deprivation.

## Introduction

It is well established, even by our own experience, that the urge to sleep increases as a function of time awake. This urge, or sleep drive, triggers a prolonged compensatory sleep after the organism is sleep deprived (Daan et al., 1984; Allada et al., 2017). This compensatory sleep, which is also called sleep rebound, is a key indicator of the homeostatic regulation of sleep (Vyazovskiy et al., 2009). In this process, deviations from a reference level of sleep are compensated, i.e., lack of sleep fosters compensatory increase in the intensity and duration of sleep, whereas excessive sleep counteracts the sleep need (Tobler and Achermann, 2007). More than a century of sleep research has made important progress in understanding the function of sleep and its regulatory circuitry, but the molecular basis of sleep homeostasis remains elusive (Sehgal et al., 2007; Cirelli and Tononi, 2008; Siegel, 2008; Donlea, 2017). Understanding the molecular mechanisms involved in the regulation of sleep homeostasis is key for the overall understanding the regulation of both the sleep circuit and the sleep function. To achieve that level of understanding, we need to study the link between molecular markers, sleep brain circuits and homeostatic sleep behavior.

The fruit fly *Drosophila melanogaster* is an ideal model to study the molecular markers impacting sleep behavior. Sleep rebound is a stable phenotype in flies which shares most major features of mammalian sleep homeostasis (Huber et al., 2004). *Drosophila* shows easily measurable and recognizable sleep patterns linked to reduced brain activity (Nitz et al., 2002; Van Swinderen et al., 2004), limited sensory responsiveness during sleep and display a robust homeostatic sleep rebound (Hendricks et al., 2000; Shaw et al., 2000) as occurs in mammals. In addition, it has been demonstrated that humans and fruit flies have a common sleep control mechanism involving GABA receptors in brain neurons linked to the circadian clock (Parisky et al., 2008; Chung et al., 2009). Moreover, fly genetics has been used as a tool to validate human sleep biomarkers affected by sleep deprivation (Thimgan et al., 2013). Hence, we circumscribed our study of the relationship between homeostatic markers and sleep behavior to the fly model.

Recent studies have shown that two structures of *Drosophila’*s brain central complex, the Ellipsoid Body (EB) and the fan body (FB), induce sleep when artificially activated, and produce insomnia, when inhibited (Donlea et al., 2011; Liu et al., 2016). Other studies have shown that neuronal microcircuits in the mushroom body (MB) drives rebound recovery after sleep deprivation (Sitaraman et al., 2015). Follow up studies have produced important progress by identifying dopamine as the neuromodulator responsible for the homeostatic switch operation between sleep/wake, which is mediated by potassium currents (Pimentel et al., 2016). Homeostatic sleep seems to be controlled by the dorsal FB neurons, which are electrically active during wake and electrically silent during rest (Pimentel et al., 2016). These studies point to the regulation of neuronal excitability as an important effector of the sleep regulation. Nevertheless, the underlying molecular framework that connects neuronal excitability with sleep behavior is a relatively unexplored area of research.

Several genes have been identified to regulate normal sleep, but only a few genes have been linked to the molecular regulation of homeostatic sleep compensation after sleep deprivation. A mutation in the *Shaker* (*Sh*) gene, which encodes a voltage dependent potassium channel involved in membrane repolarization, increases neuronal excitability and reduces normal sleep (Cirelli et al., 2005), but fails to alter sleep rebound. Interestingly, the *Shaker* activator *sleepless* (*sss*), which encodes for a brain-enriched glycosyl-phosphatidylinositol-anchored protein, impairs sleep rebound (Koh et al., 2008), perhaps by a mechanism independent of *Shaker*. The gene *crossveinless* (*cv-c*), which codes for a Rho-GTPase-activating protein, is necessary for dorsal FB neurons to transduce the excitability produced by sleep pressure into homeostatic sleep (Donlea et al., 2014). Knocking down the *Cullin 3* (*Cul3*) ubiquitin ligase gene and its putative adaptor *insomniac (inc)*, reduces sleep rebound after sleep deprivation (Pfeiffenberger and Allada, 2012). Mutants of fragile X mental retardation gene (*Fmr1*), a translational inhibitor that causes the most common form of inherited mental retardation in humans, have also been reported to reduce sleep rebound (Bushey et al., 2009). In addition, it was reported that interfering with the expression of the genes *sandman* (*sand*) and *Sh* in the dorsal FB neurons, increased or decreased sleep respectively as part of the sleep/wake switch (Pimentel et al., 2016). The regulatory picture presented by these genes and the other neuromodulators and proteins known to affect homeostatic sleep compensation seems far from complete, although together, they also point to neuronal excitability as a key component of sleep homeostatic regulation.

Unregulated neuronal excitability may lead to a potentially disruptive positive feedback. To cope with this, neurons have evolved compensatory (homeostatic) mechanisms to reduce excitability. The mechanisms by which neurons stabilize firing activity have been collectively termed “homeostatic plasticity” (Marder and Prinz, 2003; Turrigiano and Nelson, 2004; Davis, 2006; Turrigiano, 2008, 2012; Pozo and Goda, 2010). Therefore, it is plausible that wake promoting neurons, after prolonged times of wakefulness, would utilize one of the homeostatic plasticity mechanisms to regulate neuronal excitability. In this study, we begin to explore the relationship between neuronal homeostasis mechanisms and sleep regulation by testing the role of the neuronal homeostasis gene *pumilio* (*pum*) on the regulation of compensatory sleep.

The protein encoded by *pum* is characterized by a highly conserved RNA-binding domain, which acts as a post-transcriptional repressor of mRNA targets. Binding occurs through an RNA consensus sequence in the 3’-UTR of target transcripts—the Pumilio Response Element (PRE), 5′-UGUANAUA-3′, that is related to the Nanos Response Element (NRE) (Wang et al., 2018). While it was originally described in *Drosophila* for its critical role in embryonic development, Pum has an important role in the development of the nervous system. Pum is known for controlling the elaboration of dendritic branches (Ye et al., 2004), and is also required for proper adaptive responses and memory storage (Dubnau et al., 2003). Evidence of its regulatory role in neuronal excitability (Schweers et al., 2002) and homeostatic processes include Pum’s repression of translation of the *Drosophila* voltage-gated sodium channel (*paralytic*) in an activity dependent manner (Mee et al., 2004; Muraro et al., 2008). Pum-mediated repression of the voltage gated sodium channel plays a pivotal role in the regulation of neuronal homeostasis, given the central role of the sodium channel in the regulation of membrane excitability (Weston and Baines, 2007). Furthermore, Pum was found to be necessary for the homeostatic compensation of increased neuronal activity, or what is known as homeostatic synaptic depression (Fiore et al., 2014). In addition, Pum has been found to influence synaptic bouton size/number, synaptic growth and function by regulating expression of eukaryotic initiation factor 4E (eIF4E), which is the limiting factor for the initiation of the CAP dependent translation in Eukaryotes (Menon et al., 2004; Vessey et al., 2006; Cao et al., 2009). Pum was our first choice to study neuronal homeostasis effects on compensatory sleep because microarray experiments show that *pum* is expressed in Pdf-expressing neurons, which are key circadian cells known to promote wakefulness in *Drosophila* (Parisky et al., 2008; Kula-Eversole et al., 2010). With over 1000 potential targets and many others indirect targets through its eIF4E regulatory role, based on the cumulative evidence, Pum could be considered a master regulator of neuronal homeostatic processes (Menon et al., 2004; Gerber et al., 2006; Chen et al., 2008).

Studies exploring the mechanisms of neuronal homeostasis often involve long-term manipulations of neural activity, spanning from 48 h to the entire life span (Turrigiano et al., 1998; Turrigiano, 2012; Davis, 2013). Moreover, studies linking *pum* with neuronal homeostasis primarily use genetic manipulations that alter neuronal activity throughout the lifetime of the organisms (Mee et al., 2004; Weston and Baines, 2007; Muraro et al., 2008). Thus, in this study we decided to explore the role of *pum* in the regulation of compensatory sleep induced by chronic (long-term) sleep deprivation as well as acute sleep deprivation (SD).

## Results

### Knockdown of *pumilio* Abolishes Sleep Rebound After Chronic Mechanical Sleep Deprivation

We knocked down the expression of *pum* using a transgenic fly containing a *pum* RNA interference construct (*pum*^RNAi^) under control of the upstream activating sequence (UAS) of the yeast transcription factor Gal4. To activate the UAS-*pum*^RNAi^ we used a second transgenic construct that expressed Gal4 under control of the *timeless* (*tim*) gene promoter (*tim-*Gal4). When both transgenes are present in the same fly (*tim-*Gal4/UAS-*pum*^RNAi^), the *pum*^RNAi^ construct is expressed constitutively in *tim* expressing neurons. We selected the *tim*-Gal4 driver because it is a strong and broadly expressed promoter targeting circadian cells found in several brain structures including the wake promoting, Pdf-expressing ventral lateral neurons and glia (Kaneko and Hall, 2000).

**FIGURE 1:**
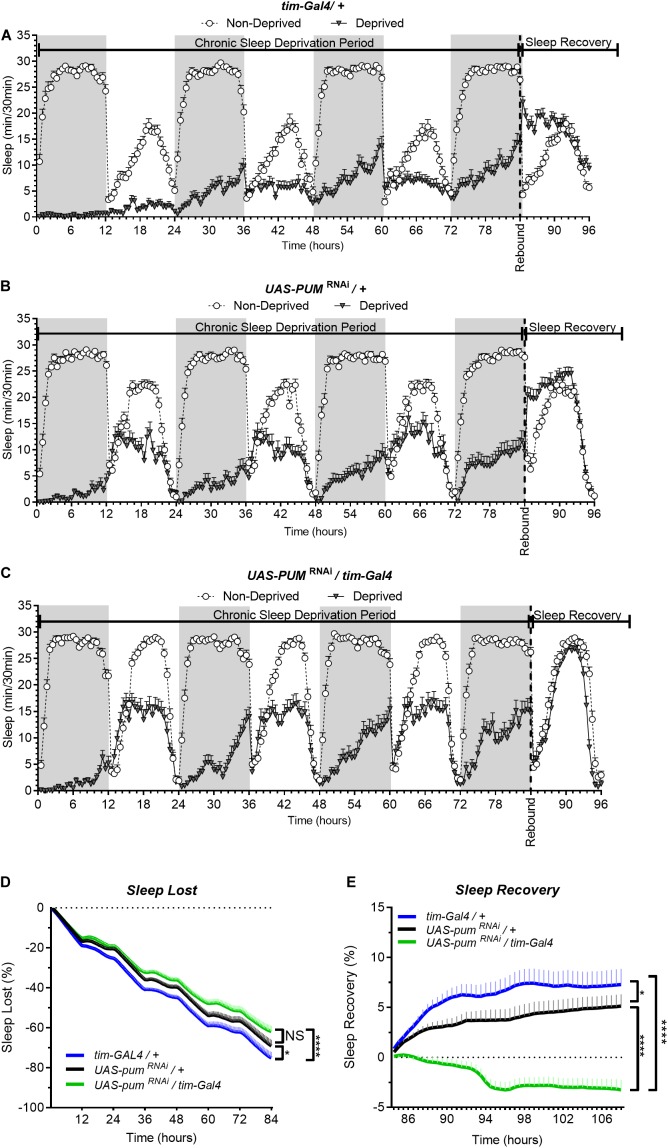
Knockdown of *pum* eliminates sleep recovery after chronic mechanical sleep deprivation. Sleep comparison of UAS*-pum*^RNAi^*/tim-*Gal4 (experimental) vs. *tim-*Gal4/+ (parental) flies and UAS-*pum*^RNAi^/+ (“sibling” controls) during chronic SD. The *X* axis indicates time after start of sleep deprivation. **(A–C)** Depiction of sleep activity during the sleep deprivation and sleep rebound period for all genotypes. **(D)** Cumulative sleep lost during deprivation expressed as a percentage of total sleep in non-deprived flies of the same genotype. Two-way ANOVA using “genotype” as a factor and “time” as a repeated measure showed significant differences in genotypes [*F*(2,132) = 11.24 *P* < 0.0001], time [*F*(167,22044) = 1033, *P* < 0.0001] and interaction [*F*(334,22044) = 3.066, *P* < 0.0001]. *Post hoc* analysis using Dunnett’s multiple comparisons test showed significant differences between *UAS-pum*^RNAi^*/tim-*Gal4 *vs. tim-*Gal4/+ flies (*P* < 0.0001). **(E)** Percent sleep recovery after SD. Two-Way ANOVA with repeat measures indicated significant differences in genotypes [*F*(2,132) = 18.58, *P* < 0.0001] and interaction [*F*(94,6204) = 13.73 *P* < 0.0001]. *Post hoc* analysis using Sidak’s multiple comparisons test comparing both control genotypes against experimental flies, revealed significant differences (*P* < 0.0001) between UAS*-pum*^RNAi^*/tim-*Gal4 *vs. tim-*Gal4/+ flies and UAS-*pum*^RNAi^/+ throughout the recovery period. The data shown represents two experiments with the following sample sizes (N): *tim-*Gal4/+ Non-Deprived (*N* = 56) and Deprived (*N* = 53); UAS-*pum*^RNAi^/+ Non-Deprived (*N* = 60) and Deprived (*N* = 35); UAS-*pum*^RNAi^/+ Non-Deprived (*N* = 63) and Deprived (*N* = 39). Because the calculations of sleep lost and sleep recovery involve both the Non-Deprived and Deprived groups (see section “Materials and Methods”), the N for panels **(D,E)** is equal to the N of the Deprived group. SD. Data points and error bars represent means ± SEM. Stars indicate significance level (*p < 0.05; ****p < 0.0001).

**FIGURE 2:**
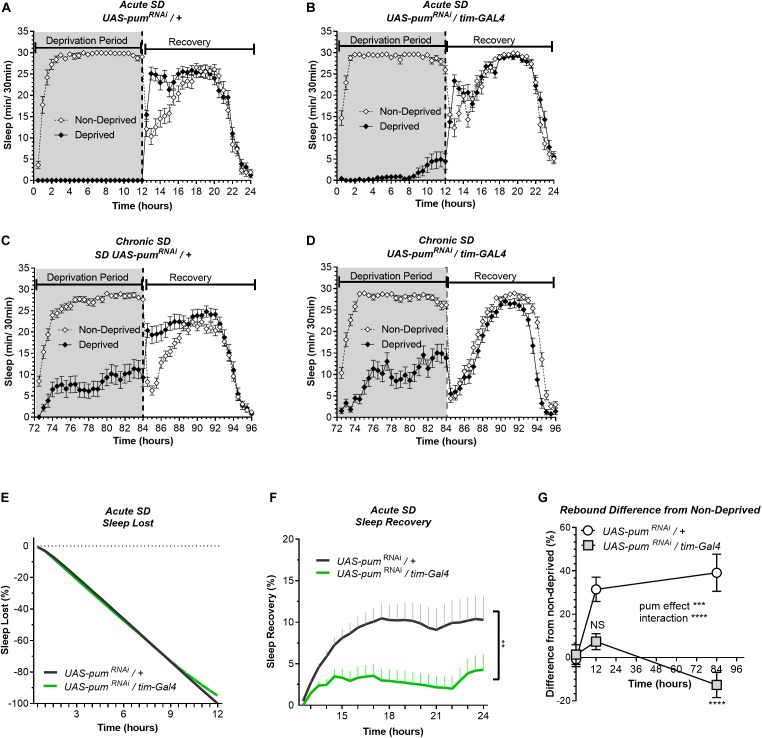
Knockdown of *pum* differentially reduces sleep recovery in acute vs. chronic SD. Sleep comparison of UAS*-pum*^RNAi^*/tim-*Gal4 (experimental) vs. UAS-*pum*^RNAi^/+ (“sibling” controls) during acute (12 h) mechanical SD. The *X* axis indicates time after sleep deprivation. **(A,B)** Depiction of sleep activity during the sleep deprivation and sleep rebound period for both genotypes during acute SD. **(C,D)** Depiction of the sleep activity during sleep deprivation and sleep rebound period for both genotypes during hours 72–96 of chronic mechanical SD included for ease of comparison. The *y*-axis shows the number of minutes that flies slept in intervals of 30 min. **(E)** Cumulative sleep lost during deprivation expressed as a percentage of total sleep in non-deprived flies of the same genotype. Two-way ANOVA, using “genotype” as a factor and “time” as a repeated measure, did not showed significant differences between the genotypes (*P* = 0.8664). **(F)** Percent sleep recovery after SD. Two-Way ANOVA with repeat measures indicated significant difference in genotypes [*F*(1,58) = 7.114, *P* < 0.0099] and interaction [*F*(23,1334) = 3.054, *P* < 0.0001]. **(G)** Percent difference in rebound between deprived and non-deprived flies after acute and chronic sleep deprivation protocols of UAS*-pum*^RNAi^/+ and UAS*-pum*^RNAi^*/tim-*Gal4 flies. Two-way ANOVA with repeated measures showed a significant difference in genotype [*F*(1,91) = 13.72, *P* = 0.0004] and time vs. genotype interaction [*F*(2,106) = 13.97, *P* < 0.0001]. *Post hoc* analysis using Tukey’s multiple comparisons test revealed significant differences between UAS*-pum*^RNAi^/+ and UAS*-pum*^RNAi^*/tim-*Gal4 at 84 h of deprivation (*P* < 0.0001) no difference was observed at 12 h (acute SD) (*P* = 0.0735). The data shown represents one experiment with the following sample sizes (N): UAS-*pum*^RNAi^/+ Non-Deprived (*N* = 31) and Deprived (*N* = 32); UAS-*pum*^RNAi^*/tim-*Gal4 Non-Deprived (*N* = 31) and Deprived (*N* = 28). Because the calculations of sleep lost and sleep recovery involve both the Non-Deprived and Deprived groups (see section “Materials and Methods”), the N for panels **(E,F)** is equal to the N of the Deprived group. Data points and error bars represent means ± SEM. Stars indicate significance level (**p < 0.01; ***p < 0.001; ****p < 0.0001).

In our first set of experiments, we subjected the parental controls (*tim*-Gal4/+), the *pum*^RNAi^ (UAS-*pum*^RNAi^/*tim-*Gal4) experimental flies and their “sibling” control flies (UAS-*pum*^RNAi^/+), which carry the *pum*^RNAi^ construct by itself and has a closer genetic similarity to the experimental flies than parental controls, to a chronic (84 h) mechanical SD protocol (see section “Materials and Methods”). The results from the chronic SD showed a strong effectiveness of the sleep deprivation method during the first 12 h (Figure 1A). As time progressed, we noticed a gradual increase in the amount of sleep in all the sleep deprived genotypes during sustained mechanical deprivation (Figures 1A–C), which we first though could be an adaptation to the SD method. However, this increase in sleep through time did not seem to affect the sleep rebound, as both parental and “sibling” control flies were able to produce a normal sleep rebound pattern that initiated at the 84th hour—immediately after the SD protocol was terminated (Figures 1A,B). If we look more closely at the recovery period in Figure 1C (which could be better viewed in Figure 2D that has the *x* axis expanded), we can see that sleep-deprived flies with silenced *pum* are more active than non-deprived flies. This difference becomes more obvious in the sleep recovery plot (Figure 1E), where sleep levels are normalized against the non-deprived flies, and it is evident that flies with silenced *pum* begin the sleep recovery period close to zero percent but slowly decreased with time. This tendency continued decreasing for the next 48–72 h until it stabilized (Supplementary Figure S2). To determine if this lack of sleep rebound was related to adaptation to the SD method leading to insufficient sleep deprivation, we quantified the sleep loss of all experimental groups (Figure 1D). This quantification of cumulative sleep loss during the 84-h deprivation period showed a significant difference between the *pum*^RNAi^/*tim-*Gal4 flies and the *tim-*Gal4/+ parental control flies, but no difference between the *pum*^RNAi^/*tim-*Gal4 flies and the UAS-*pum*^RNAi^/+ “sibling” controls (Figure 1D). The fact that this difference was not significant between the genetically closer “sibling” control and experimental *pum*^RNAi^ flies, suggests the difference in deprivation effectiveness between parental controls and *pum*^RNAi^ flies could be due to an adaptation to the SD method influenced by the genetic background. We used this sleep lost value to normalize the sleep recovery calculation (see section “Materials and Methods”) (Figure 1E). The results for sleep recovery show a normal recovery pattern, as indicated by the increase in cumulative sleep recovered during the first hours after SD. This result is indicative of compensatory sleep present in both parental and “sibling” controls after sleep deprivation (Figure 1E). However, *pum*^RNAi^ flies showed a negative sleep recovery, which indicates *pum*^RNAi^ flies were even more active than non-deprived flies after 84 h. of continuous deprivation (Figure 1E). This loss of homeostatic regulation in the recovery of *pum*^RNAi^ flies was maintained up to 96 h post-deprivation with no mortality in any of the groups after 108 h post deprivation (see Supplementary Figure S3).

In our experiments, the UAS-*pum*^RNAi^/+ control lines are “siblings” of the UAS-*pum*^RNAi^/*tim-*Gal4 flies. Meanwhile the *tim-*Gal4/+ controls were generated directly by crossing the parental *tim*-Gal4 line with a non-transgenic wild type (CS), which can introduce differences in genetic background. Thus, our conclusions are based mostly on the results from “sibling” controls because they have a greater genetic similarity, which results in a more similar baseline sleep pattern than parental controls (Figures 1A–C). Hence, for the following acute SD experiments, parental controls were not used.

### Pumilio Regulates Sleep Rebound Differentially Between Acute and Chronic Mechanical Sleep Deprivation

The results from the 12 h acute SD showed sleep lost effectivity close to 100% for both *pum*^RNAi^ and “sibling” controls (Figures 2A,B). During the deprivation period (0–12 h), the cumulative sleep loss in deprived flies did not show a significant difference between the two genotypes (Figure 2E). Once again, controls showed an effective sleep rebound (Figure 2A), while *pum*^RNAi^ flies showed a reduction in sleep rebound (Figure 2B). However, this time the rebound was not completely abolished as we observed during chronic SD (Figure 2B vs. Figure 2D). We included the chronic deprivation rebound period as a point of comparison between acute vs. chronic (Figures 2C,D). The results from the acute SD sleep recovery resembled the results from chronic SD with a normal rebound in “sibling” controls and reduced sleep recovery in *pum*^RNAi^ flies. Nevertheless, the sleep recovery of *pum*^RNAi^ flies was not negative as we observed during chronic SD (Figure 2F). When acute vs. chronic SD results are compared (Figure 2G), we see significant differences, not only between the genotypes, but also within *pum*^RNAi^ flies exposed to acute vs. chronic SD, while the rebound difference of the “sibling” control between acute vs. chronic SD remains constant. These results suggest that *pum* differentially regulates acute vs. chronic SD. This interpretation is in fact reinforced by our molecular experiments contrasting gene expression changes between acute and chronic SD as reported in the Supplementary Figure S5.

So far, our findings link the duration of sleep deprivation to increased *pum* regulation, which is consistent with our expectations. Since we observed greater homeostatic changes during chronic SD, we continued throughout the study using only chronic SD to measure *pum*’s regulatory effects in compensatory sleep. The difference in sleep rebound between *pum*^RNAi^ vs. parental flies does not seem to be related to non-specific effects of the genetic background affecting baseline sleep because daytime baseline sleep of *pum*^RNAi^ flies is higher than both parental and “sibling” controls (Supplementary Figure S2). If baseline sleep would have been a contributing factor for the recovery results, we should have expected a higher sleep rebound, not lower. The fact that we obtained a lower rebound indicates a Pum knockdown effect rather that genetic differences influencing baseline sleep are the culprit of our results.

### *Pumilio* Mutants Show Reduced Sleep Rebound

To confirm the effects of *pum* knockdown in sleep homeostasis independently of transgenic flies, we tested mutant fly lines to further validate our results. First, we selected the classical loss of function allele *pum*^13^ (also known as *pum*^680^). *Pum*^13^ is a dominant negative allele that bears a single amino acid substitution, which not only knocks down Pum function but also interferes with normal Pum function in heterozygotes (Wharton et al., 1998). Thus, in addition to the semi-lethal *pum*^13^ homozygous mutants, we used *pum*^13^/*TM3* heterozygotes in our experiments.

**FIGURE 3:**
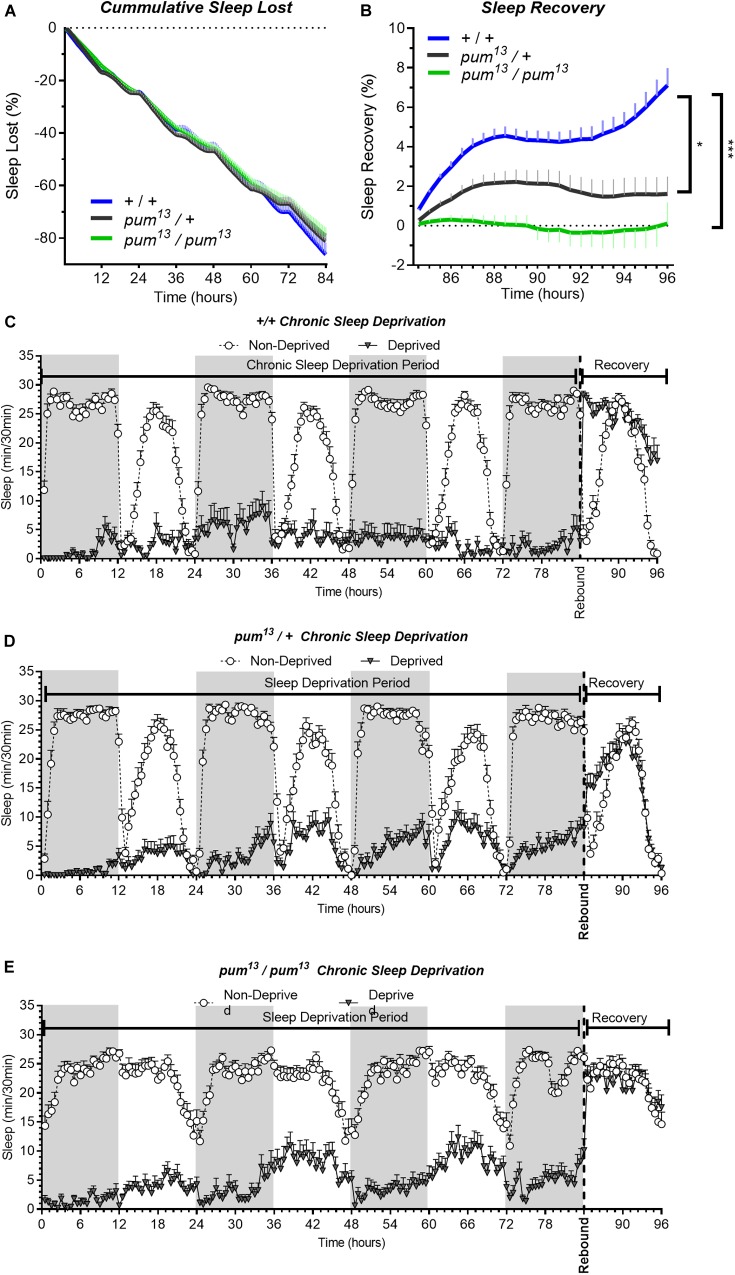
*pum*^13^ mutant show reduced sleep rebound after chronic SD. Sleep comparison of wild type, heterozygous and homozygous flies for the *pum*^13^ allele. **(A–C)** Depiction of sleep activity during the sleep deprivation and sleep rebound period for all genotypes. The *X* axis indicates time after the start of the sleep deprivation protocol. The *y*-axis shows the number of minutes that flies slept in intervals of 30 min. **(D)** Cumulative sleep lost during deprivation expressed as a percentage of total sleep in non-deprived flies of the same genotype. Two-way ANOVA, using “genotype” as a factor and “time” as a repeated measure, did not show significant differences between the genotypes [*F*(2,63) = 0.3635, *P* = 0.6967]. **(E)** Percent sleep recovery after SD. Two-Way ANOVA with repeat measures indicated significant difference in genotypes [*F*(2,63) = 11.29, *P* < 0.0001] and interaction [*F*(46,1449) = 5.667, *P* < 0.0001]. *Post hoc* analysis using Uncorrected Fisher’s LSD comparisons test comparing all genotypes against *pum*^13^/*pum*^13^ flies revealed significant differences with *pum*^13^/+ flies (*P* = 0.0319) and with *pum*^13^/*pum*^13^ (*P* < 0.0001). The comparison between *pum*^13^/+ and *pum*^13^/*pum*^13^ show no difference (*P* = 0.0728). The data shown represents one experiment with the following sample sizes (N): (1) Canton-S (+/+), Non-Deprived (*N* = 30) and Deprived (*N* = 17); *pum*^13^/+, Non-Deprived (*N* = 28) and Deprived (*N* = 28); *pum*^13^/*pum*^13^, Non-Deprived (*N* = 30) and Deprived (*N* = 22). Because the calculations of sleep lost and sleep recovery involve both the Non-Deprived and Deprived groups (see methods), the N for panels **(A,B)** is equal to the N of the Deprived group. Data points and error bars represent means ± SEM. Stars indicate significance level (*p < 0.05; ***p < 0.001).

The sleep deprivation produced similar sleep lost amounts in each of the lines tested. Figures 3A–C,D). Nonetheless, the sleep recovery showed a significant difference between both wild type (+/+) and *pum^13^/*+flies compared to *pum*^13^/*pum*^13^ flies (Figure 3E). By the end of the recovery period, the differences between *pum^13^/*+ and the knockout *pum^13^/pum^13^* were still maintained. Moreover, *pum*^13^/*pum*^13^ escaper flies completely abolished rebound to chronic sleep deprivation for the first 12 h of the recovery period (Figure 3E). This suggests that differential *pum* levels between the heterozygote and the *pum*^13^ homozygote, have correlative regulatory effects in sleep rebound.

**FIGURE 4:**
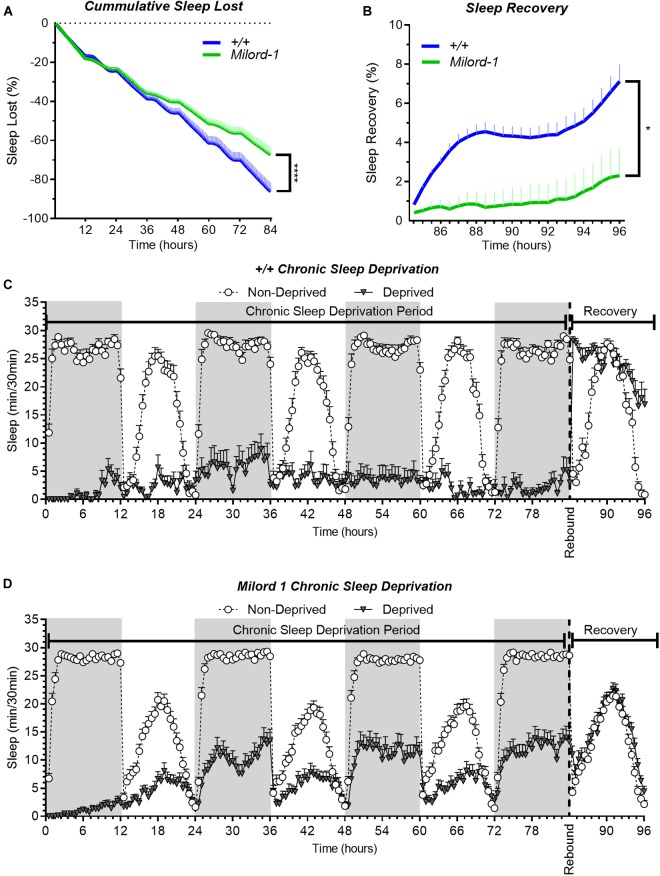
The Milord-1 fly line shows reduced sleep rebound after chronic SD. Sleep comparison of wild type and Milord-1 flies. **(A,B)** Depiction of sleep activity during the sleep deprivation and sleep rebound period for all genotypes. The *X* axis indicates time after the start of the sleep deprivation protocol. The *y*-axis shows the number of minutes that flies slept in intervals of 30 min. **(C)** Cumulative sleep lost during deprivation expressed as a percentage of total sleep in non-deprived flies of the same genotype. Two-way ANOVA using “genotype” as a factor and “time” as a repeated measure showed no significant differences between the genotypes [*F*(1,58) = 3.712, *P* = 0.0589]. **(D)** Percent sleep recovery after SD. Two-Way ANOVA with repeat measures indicated significant difference in genotypes [*F*(1,58) = 5.193, *P* = 0.0264] and interaction [*F*(23,1334) = 1.695 *P* < 0.0213]. The data shown represents two experiments with the following sample sizes (N): Canton-S (+/+) Non-Deprived (*N* = 30) and Deprived (*N* = 17); Milord-1 Non-Deprived (*N* = 62) and Deprived (*N* = 45). Because the calculations of sleep lost and sleep recovery involve both the Non-Deprived and Deprived groups (see methods), the N for panels **(A,B)** is equal to the N of the Deprived group. Data points and error bars represent means ± SEM. Stars indicate significance level (*p < 0.05; ****p < 0.0001).

Additionally, we used the p-element insertion *pum* allele, Milord-1, to confirm the mutant results with another independent line. This line was generated by single transposon mutagenesis inserted in the *pum* transcriptional unit (Dubnau et al., 2003). We compared this line with controls obtained from a wild type stock Canton S flies. As expected, Milord-1 flies showed a significant sleep rebound reduction (Figure 4D). Although there was a significant sleep lost difference between the genotypes at the end of the deprivation period (Figure 4C), the ANOVA table results did not show a significant difference between the genotypes for the whole deprivation period. In addition, the sleep recovery calculation normalizes by the sleep lost, therefore, any sleep lost differences affecting the results have already been considered.

### *Pumilio* Knockdown in Tim Neurons Increases the Number of Pdf Puncta

In order to directly observe the effects of *pum* knockdown in the *Drosophila* brain, we selected the small ventral Lateral Neurons (sLNv) which are an important part of the circadian wake promoting system in *Drosophila* (Parisky et al., 2008). This subset of *tim* positive neurons (Seluzicki et al., 2014) are characterized by the secretion of the neuropeptide Pigment Dispensing Factor (Pdf). We wanted to evaluate the effect of *pum* knockdown on the morphology of these neurons firstly because it has been reported that flies lacking *pum* exhibit abnormalities in dendrite morphology (Ye et al., 2004) and secondly because Pdf secretion have been shown to increase during sleep deprivation in *Drosophila* neurons (Bushey et al., 2011). Therefore, we should expect to see an overall increase in Pdf immunofluorescent signal as a result of the sleep deprivation method in sleep deprived flies.

**FIGURE 5:**
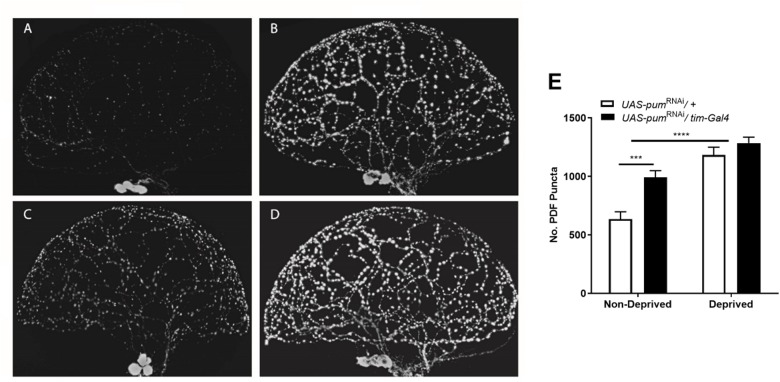
Knockdown of *pum* flies showed increased Pdf neurons puncta and pdf expression after chronic sleep deprivation. **(A–D)** Representative confocal images of anti-pdf immunofluorescence of *Drosophila* brain optic lobes showing Pdf neurons terminals. All panels were adjusted to the same gain intensity. **(A)** Representative immunofluorescence for UAS-*pum*^RNAi^/+ non-deprived “sibling” controls (*n* = 10). **(B)** Representative immunofluorescence for UAS-*pum*^RNAi^/+ deprived “sibling” controls (*n* = 12). **(C)** Representative immunofluorescence for UAS-*pum*^RNAi^/*tim*-Gal4 non-deprived experimental flies (*n* = 12). **(D)** Representative immunofluorescence for UAS-*pum*^RNAi^/*tim*-Gal4 deprived experimental flies (*n* = 9). **(E)** Quantification of Pdf neurons puncta from anti-Pdf immunofluorescence labeled *Drosophila* brain lobes. A Two-way ANOVA shows a significant interaction [*p* = 0.0438, *F*(1,39) = 4.339] and significant differences between the factors deprivation [*p* = 0.0006, *F*(1,39) = 14.04] and genotype [*p* < 0.0001, *F*(1,39) = 47.55]. *Post hoc* using Tukey’s multiple comparisons test showed differences within genotypes for deprived vs. non-deprived flies. Stars indicate significance level (***p < 0.001; ****p < 0.0001).

For illustration purposes, we selected 4 representative brain lobes from each of the 4 experimental groups in the study: non-deprived “sibling” controls (Figure 5A), deprived “sibling” controls (Figure 5B), non-deprived *pum*^RNAi^ (Figure 5C), and deprived *pum*^RNAi^ flies (Figure 5D) and adjusted all the images to the same gain. We did not observe morphological abnormalities in any of the groups. The effect of *pum* knockdown in *pum*^RNAi^ flies could be clearly observed in both the fluorescence intensity and the number of Pdf puncta by comparing non-deprived “sibling” controls vs. non-deprived *pum*^RNAi^ flies (Figure 5A vs. Figure 5C). Quantification from anti-Pdf immunofluorescence throughout the sLNv arbor in both the genetic “sibling” control and *pum*^RNAi^ flies, based on counting Pdf-reactive (Pdf+) puncta, showed that *pum*^RNAi^ non-deprived flies had a significant increase in Pdf puncta when compared with non-deprived controls (Figure 5E). Nevertheless, we did not see a significant increase in deprived *pum*^RNAi^ flies vs. deprived “sibling” controls (Figure 5D vs. Figure 5B) perhaps due to some ceiling effect when the increase observed in *pum*^RNAi^ flies (Figure 5A vs. Figure 5C) is combined with the increase seen during deprivation (Figure 5A vs. Figure 5B). These results indicates that *pum* knockdown has a direct effect circadian Pdf wake promoting neurons, without affecting their morphology.

## Discussion

Through the behavioral data of transgenic RNAi knockdowns and mutant behavioral analysis, our results indicate that *pum* is necessary for the compensatory sleep behavior displayed after sleep deprivation in *Drosophila.* The *pum-*dependent regulation of sleep compensation increases as sleep needs increases as demonstrated by the sleep rebound differences between acute vs. chronic SD, together with the differential gene expression patterns. Compensatory sleep rebound after a 12-h sleep deprivation protocol (acute SD) was slightly reduced by knockdown of *pum* in *tim* neurons, but completely abolished after 84-h of sleep deprivation (chronic SD).

Interestingly, we also observed that *pum*^RNAi^ flies have increased day-time sleep in non-deprived conditions (Figure 1 and Supplementary Figure S2A), suggesting that other sleep behaviors are also regulated by *pum.* This effect of *pum* could perhaps be explained by the increased expression levels of *Gad1* and *Shal* in *pum*^RNAi^ non-deprived flies, as both genes are associated with a depression in overall neural activity (Supplementary Figure S5A). Additionally, the role of *pum* on regulating baseline sleep seems to be disconnected from its role in regulating sleep rebound. For instance, the daytime baseline sleep, in *pum*^RNAi^ flies is about two times the baseline of both control flies (Supplementary Figure S2A), but the same flies showed no rebound sleep after SD, suggesting that the homeostatic sleep rebound is independently regulated from baseline sleep. This interpretation is supported by reports from other groups. Shaw et al. (2002) previously reported that *cycle* (*cyc01*) mutants showed an exaggerated response to sleep deprivation, which was 3 times as high as baseline sleep. In addition, Seidner et al. (2015), found evidence suggesting that baseline sleep and homeostatic sleep are regulated by distinct neural circuits, which has been also independently corroborated by other studies (Liu et al., 2016). As reported by Seidner et al. (2015), thermo-activation of selected cholinergic neurons activates the sleep homeostat to promote rebound, whereas thermo-activation of octopaminergic neurons suppresses the sleep homeostat and produce “negative rebound.” In this context, a potential explanation for our finding that flies with *pum* silenced in the *tim*-Gal4 circuit exhibited a “negative rebound” after chronic sleep deprivation is that the processes that promote and suppress sleep homeostasis are recruited by our deprivation protocol. In the presence of Pum the homeostatic promoting process dominates, whereas when Pum is reduced, the homeostatic suppressing process dominates.

It is important to note that the *tim*-Gal4 driver used in these experiments has broad expression pattern, which is not limited to circadian neurons, and includes non-circadian neurons as well as glial cells. Interestingly, recent studies have pointed to a possible role of glia in the homeostatic control of sleep (Krzeptowski et al., 2018). Thus, in our view, including both clock neurons and glia in our analysis of sleep homeostasis is a reasonable choice to use as a primary probe. Restricting our manipulation to just neurons (as with *elav*-Gal4) or to only specific clock neurons may have precluded a positive result. Furthermore, *tim*-Gal4 expression is very strong in adults compared to other drivers. In fact, *tim* expression during development is highest in the adult brain and lowest during the developing embryo and larva. In contrast, *elav*-Gal4 has very high expression levels during development, but relatively low adult expression. While our choice certainly leaves the precise anatomical location of Pum’s effects in sleep rebound undefined, it has narrowed down the possibilities for future research.

As an RNA-binding protein regulating the translation of many target genes, Pum regulates many biological processes. Among these processes some could be associated with the results presented in this study. These include the regulation of neuronal excitability, glial function, Epidermal Growth Factor Receptor (EGFR) signaling, inflammatory pathways, the cell cycle gene network, and neural development. With regards to neuronal excitability, a number of studies have shown that neural plastic changes that increase the excitability of specific sleep-promoting neurons are fundamental for encoding sleep pressure (Donlea et al., 2014; Sitaraman et al., 2015; Liu et al., 2016; Guo et al., 2018; Ni et al., 2019). In contrast to sleep-promoting neurons, wake-promoting neurons exhibit decreased neuronal excitability after deprivation and this change is also associated with the generation of sleep pressure (Sitaraman et al., 2015). Since Pum is known to decrease neuronal excitability under a wide range of experimental conditions (Schweers et al., 2002; Mee et al., 2004; Driscoll et al., 2013; Lin and Baines, 2015), a potential mechanism is that Pum contributes to sleep homeostasis in the following manner: (1) participating in the generation of sleep pressure by decreasing the excitability of wake-promoting neurons during deprivation and (2) participating in the dissipation of sleep pressure after sleep deprivation by decreasing the excitability of sleep promoting neurons during rebound. In addition, a network of cell cycle genes has recently been implicated in sleep homeostasis through a post-mitotic function, presumably by regulating the excitability of the Pars Lateralis (PL) neurons by the CDK1 kinase (Afonso et al., 2015). Interestingly, a regulator of the CDK1 kinase, CycB, is a known target of Pum. Aside from the lack of experimental evidence directly testing these hypotheses, other limitations of these interpretations are that the main Gal4 line used in this study, *tim*-Gal4, has little or no expression in some of the main sleep homeostasis centers (Ellipsoid Body, dorsal Fan Shape body, Mushroom Body and Pars Lateralis) (Kaneko and Hall, 2000; Rogulja and Young, 2012), and that the role of circadian neurons on sleep homeostasis is just beginning to be elucidated (Guo et al., 2018; Lamaze et al., 2018; Ni et al., 2019).

Another site of convergence between biological processes affected by Pum and sleep homeostasis is the glial cells. For instance, Pum directly interacts with various components of the Notch signaling pathway in mammals, and it has been shown that glial Notch signaling in *Drosophila* negatively regulates sleep homeostasis (Seugnet et al., 2011). Importantly, Pum is expressed in *Drosophila* glial cells (Ng et al., 2016) and *tim*-Gal4 expression is very strong in these cells (Kaneko and Hall, 2000). The role of glial cells on sleep homeostasis is slowly becoming clearer and has been shown in both flies and mammals (Halassa et al., 2009; Chen et al., 2015; Vanderheyden et al., 2018, 2019; Frank, 2019). A key role of mammalian Pum in reactive astrogliosis after brain injury has also been described, which further strengthens the idea that this gene plays an important role in glial cell function (Kanemaru et al., 2013).

Other possible Pum functions that could have played a role in our results include EGFR and the regulation of inflammatory pathways. EGFR is a direct target of Pum in *Drosophila* and has been implicated in sleep regulation in flies, worms, zebrafish and humans (Foltenyi et al., 2007; Kim et al., 2012; Lee et al., 2019; Konietzka et al., 2020). These studies showed that EGFR signaling promotes sleep in all organisms tested but its role in sleep homeostasis was only examined in zebrafish. Although it is clear that Pum suppresses EGFR signaling in *Drosophila*, the sleep homeostasis effects observed in this study are unlikely due to EGFR regulation since Foltenyi et al. (2007) tested various circadian drivers, including the *tim*-Gal4 driver, and did not observe any effect of EGFR signaling manipulations with these Gal4 lines. Regarding the contribution of Pum’s inflammatory pathways regulation to our results, a genome-wide identification of mRNAs carrying Pum binding sites, found that 27% of Pum-regulated genes were immunity genes including various antimicrobial peptides and cytokines (Gerber et al., 2006). Cytokines are increased in chronically sleep deprived patients and a number of them have been shown to be sleep-regulatory substances (SRSs) mediating sleep homeostasis (Krueger, 2008; Allada et al., 2017; Nguyen et al., 2019; Tan et al., 2019). Gila cells and plasmatocytes are a major source of cytokines; therefore, these potential Pum effects cannot be ruled out.

The anatomical loci for Pum effects on sleep homeostasis remains unclear but based on the rapidly growing literature on the cellular and molecular bases of sleep homeostasis, we can speculate potential cellular groups. Accumulating evidence indicates that although there are many wake and sleep promoting cellular groups in the brain, only a small fraction participates in the homeostatic control of sleep. For instance, Seidner et al. (2015) screened 374 randomly selected Gal4 drivers from the Janelia Farm library. Interestingly, they found that while 10% were robust wake-promoting drivers, only 11% of those, which represent 1% of the total, exhibited a sleep homeostatic response. Within the expression pattern of the *tim*-Gal4 driver used in this study, the only cellular groups that have been directly linked to sleep homeostasis regulation are a subset of the dorsal neurons (the DN1p), the lateral posterior neurons (LPNs) and glial cells (Kaneko and Hall, 2000; Seugnet et al., 2011; Chen et al., 2015; Guo et al., 2018; Hsu and Sehgal, 2018; Lamaze et al., 2018; Vanderheyden et al., 2018, 2019; Ni et al., 2019). Although core clock neurons such as the Pdf cells and the lateral neurons dorsal group (LNd) have been shown to be wake-promoting, their role on sleep homeostasis has not been directly described. Pivotal neurons in the control of sleep homeostasis such as those in the ellipsoid bodies (EB) and fan-shape body (FB) are not included in the *tim*-Gal4 circuit. However, these regions are surrounded by *tim*-positive glial cells (Kaneko and Hall, 2000). A brain region that has undetectable Tim protein levels but has strong expression in the *tim*-Gal4 driver is the sub-esophageal ganglion (SEG). This region is relevant in this context since the set of wake-promoting neurons with the strongest influence on sleep homeosis described, are cholinergic neurons that reside on this region (Seidner et al., 2015).

Although our chronic sleep deprivation protocol is very effective during the first 12 h of deprivation, we consistently observed a gradual sleep increase in all transgenic and mutant lines tested. The degree of this adaptation varies greatly among the different lines and is not consistently associated with *pum* knockdown. Comparisons between UAS-*pum*^RNAi^/+ and UAS-*pum*^RNAi^/*tim*-Gal4 line (Figure 1), or *pum*^13^/+ and pum^13^/pum^13^ (Figure 4), did not show significant differences in sleep lost. The only comparison showing a significant difference in sleep lost during the deprivation period is the wild type Canton S flies vs. Milord-1 allele. This is in great contrast with the sleep rebound phase, in which all comparisons consistently show a reduction on sleep rebound. Even though there is not an association with *pum* manipulation, we believe that this adaptation is a sleep homeostatic response since we have consistently observed in wild type flies that individuals that are able to sleep during the deprivation period, show reduced sleep rebound compared to individuals more effectively deprived (data not shown).

Our finding that *pum* knockdown had a more dramatic effect after chronic SD compared to acute SD indicates that *pum’s* control of translation becomes progressively more important with deprivation time. Moreover, this suggests that sleep homeostasis involves a gradual recruitment of different processes to achieve its homeostatic goal depending on the chronicity of the sleep depriving insult. Consistent with this idea, Liu et al. (2016) pointed out that the short half-life’s (minutes) of the sleep-regulatory substances (SRSs) thought to underlie sleep pressure, was inconsistent with the time course for dissipating sleep pressure (hours or even days). Furthermore, they showed that neural plastic changes in specific circuits within the ellipsoid body are necessary and sufficient for generating sleep pressure. Initial studies of chronic SD in other species have also pointed to a potential difference in the regulatory mechanisms between acute vs. chronic SD. Rats exposed to chronic SD do not seem to regain the sleep lost even after a full 3-day recovery period, whereas in acute deprivation, most of the sleep was regained (Kim et al., 2007). Critics attributed these differences, between acute and chronic SD, to the increase in sleep pressure, which force micro-sleep episodes or EEG artifacts during chronic SD (Leemburg et al., 2010). A more recent study showed that chronically sleep deprived animals no longer express the compensatory increases that characterize sleep homeostasis in daily sleep time and sleep intensity (Kim et al., 2013). The authors of the study suggested that this decoupling of sleepiness from sleep time/intensity imply that there is one sleep regulation system mediating sleepiness (homeostatic), and another regulatory system for sleep time/intensity (allostatic) (Kim et al., 2013). Whether the lack of sleep compensation observed during chronic SD is a real mechanistic phenomenon or an artifact of the deprivation method remained controversial. In our study, we wanted to test if the behavioral differences reported by the literature, between acute and chronic SD, were regulated by the same mechanism under the *pum* gene. Our results point to the presence of a differential homeostatic response between acute vs. chronic SD in *pum* knockdowns, which suggests that *pum* participation in sleep homeostatic regulation is proportional to sleep need. Our data also suggests that *pum* regulation of sleep rebound is done through differential gene activation between acute and chronic SD. This difference seems to be aligned with fast action ion channel genes for acute SD and translation related and/or genes in which we expect to require more time to become active for chronic SD.

Another consideration is that the decrease in sleep rebound observed during *pum* knockdown is associated an increase in fly activity, which is related to increased neuronal excitability (Tabuchi et al., 2015). One possible explanation for these results is Pum known regulation of sodium currents (Ina) and excitability in *Drosophila* motor neurons through translational repression and binding with *para*-RNA (Baines, 2003; Driscoll et al., 2013). Reducing *pum* expression means there could be more sodium channels available and consequently, more neurons excited. Those excited neurons would have a diminished homeostatic mechanism to couple with the increase in excitability, resulting in prolonged wakefulness even after sleep deprivation stimulus was discontinued. Additional evidence in the literature supports the notion of a direct correlation between ion channels activity and wakefulness. Parisky et al. (2008), expressed the EKO potassium channel to hyperpolarize Ventral Lateral neurons (LNv) to reduce their excitability. In addition, they knocked down the *Shaw* potassium channel gene or expressed a dominant-negative Na+/K+-ATPase α subunit in the Pdf LNv neurons in order to increase neuronal excitability. The results showed that suppressed LNvs increased sleep whereas hyperactive LNvs increased wake. Furthermore, studies in rats have shown increases in cortical neurons firing with an increase in time awake (Vyazovskiy et al., 2009). Moreover, Donlea et al. (2014) found that the *crossveinless (cv-c)* mutants show decreased electrical activity in sleep promoting dorsal fan neurons. Additionally, the same study found that sleep pressure increases electrical excitability of sleep promoting neurons and this mechanism was blunted in *cv-c* mutants. This favors the alternative that *pum* regulates sleep homeostasis through the regulation of neuronal excitability.

Gene expression studies of *pum* mRNA levels in heads of *pum*^RNAi^ flies and their respective “sibling” controls indicated that the RNA interference leads to a 50% reduction in non-deprived flies. Nevertheless, when we examined *pum* mRNA levels in sleep-deprived animals, we found a dramatic increase in *pum*^RNAi^ flies and their respective “sibling” controls. Although this is a very counterintuitive result, we speculate that this is probably the result of a sleep-deprivation-induced increase in *pum* levels in cells outside the *tim*-Gal4 circuit. Furthermore, this finding is consistent with the idea that the “negative rebound” observed in *pum*^RNAi^ flies, but not in *pum*^13^ and Milord-1 hypomorphs, is due to *pum* actions in cells outside the *tim*-Gal4 circuit.

Finally, our analysis of gene expression presented in the Supplementary Material indicates that *pum* knockdown in *tim* cells induces a series of global changes in gene expression that may contribute to the lack of homeostatic sleep response. However, this analysis suffers from major limitations. Because the qRT-PCR analysis uses RNA extracted from whole heads, which include many more cells than covered by the driver line used to knockdown *pum*, it is difficult to interpret the results. Small changes in expression in *tim* cells will likely be lost in noise, whilst larger changes may persist. Furthermore, we do not know whether the changes in mRNA are happening within *tim* cells or somewhere else. Thus, we cannot infer a direct role of *pum* in any of the changes in mRNA observed. Nonetheless, one striking observation is that, in general, the expression levels of most genes tested remains relatively stable upon acute and chronic sleep deprivation (<2-fold change). However, when *pum* is knocked down specifically in *tim* cells, global expression of the same genes is dramatically altered upon deprivation (4 to 16-fold change). This suggests that Pum is necessary, either directly or indirectly, in keeping these genes in check after deprivation. Another important observation is that in most cases the genes affected by Pum knockdown after acute deprivation are not the same as those affected after chronic deprivation. This suggests that the two responses are controlled, at least partially, by distinct mechanisms.

In summary, our results indicate that Pum is necessary for a normal sleep rebound after sleep deprivation. In addition, we showed that this effect is more pronounced during chronic sleep deprivation (84 h) than acute deprivation (12 h). These behavioral changes were associated with accompanying differential changes in the expression of genes involved in synaptic translation and the regulation of neuronal excitability. Based on these findings, we propose that Pum is an important regulator of sleep homeostasis through neural adaptations triggered during sleep deprivation and induces rebound sleep. Further studies characterizing additional Pum targets warrant exciting findings about the molecular control of sleep. Moreover, future studies identifying the specific circuits where Pum is required for sleep regulation could provide a better picture of the mechanistic relationship between sleep function and molecular sleep regulation.

## Materials and Methods

### Fly Stocks

*Drosophila* stocks were raised on standard *Drosophila* medium in a 12/12 h light/dark cycle. The following stocks were used in this study: The UAS*-pum*^RNAi^ (stock #26725: y[1] v[1]; P{y[+t7.7] v[+t1.8]=TRiP.JF02267}attP2) fly line was obtained from Bloomington Stock Center; The *tim*-Gal4 transgenic line: *yw; cyo/tim-*Gal4 was obtained from Dr. Leslie Griffith’s and Dr. Michael Rosbash’s labs at Brandeis University. These two lines were crossed to obtain both UAS-*pum*^RNAi^/*tim-*Gal4 experimental flies and the “sibling” control flies UAS-*pum*^RNAi^/+. The Milord-1 P{lacZ}^pummilord–1^ was obtained from Dr. Josh Dubnau. The mutant *pum*^13^ (*pum*^680^) and Canton S wild type flies were also obtained from Bloomington Stock Center and crossed to obtain both *pum^13^/* + and *pum*^13^/*pum*^13^ flies used in Figure 5.

### Sleep Assays

Sleep assays used 1–2 days old female flies. The individuals were collected, separated by phenotype and placed into controlled temperature for 6–7 days under 12 h:12 h light dark cycles for entrainment. The individuals were then anesthetized with CO_2_ and placed in individual tubes containing fly food (5% sucrose, 2% agar). Tubes were then placed in *Drosophila* Activity Monitors (DAM) within an environmentally controlled incubator (26°C, 80% humidity, light intensity of 800 lux) and connected to the monitoring system (TriKinetics, Waltham, MA, United States) under 12 h:12 h light dark cycles. After 6 days of baseline recordings, after changing the fly food to avoid dryness and microbial growth, the different groups of flies were sleep deprived with the methods described below. The genetic controls (“siblings”) were handled and tested side by side to the experimental flies. Flies with less than 80% deprivation within the first 12 h were excluded from the analysis. Number of individuals tested and number of experiment replications depicted are stated in figure legends. A cumulative sleep lost plot was produced by calculating the cumulative sleep difference between the deprivation period and the baseline period before sleep deprivation. The individual sleep recovery (rebound) was calculated by dividing the cumulative amount of sleep regained by the total amount of sleep lost during deprivation.

### Mechanical Sleep Deprivation

Mechanical deprivation was performed using a commercially available *Drosophila* sleep deprivation apparatus (Trikinetics Inc., VMP Vortexer Mounting Plate). The apparatus was controlled by the Trikinetics software, shaking the monitors for 30 s on alternate settings of 4, 5, and 8 min to create an apparently random shaking pattern. The same pattern was used for all experiments. The flies were placed in the *Drosophila* Activity Monitors to be monitored for 6 days for baseline sleep. After the 6th day, flies were subjected to mechanical SD. Both chronic and acute deprivation protocols were identical in terms of stimulus intensity and pattern; the only difference was the duration of the deprivation period. For chronic sleep deprivation, the SD protocol was active for the first 84 h starting at the beginning of the first dark period (Figure 1), while for acute sleep deprivation, the SD protocol lasted only 12 h, which encompassed the entirety of the dark period preceding the sleep recovery period. For the acute SD experiment, the same set up was used but for only 12 h of the deprivation night. Although this protocol results in partial sleep deprivation, rather than total deprivation, it induces significant sleep lost, normally around 80%, and allows the flies to survive through the chronic sleep deprivation period. Due to the long SD time of 84 h and the baseline period, we perform a fly food change the day before SD to avoid microbial growth and food dryness. This change is coordinated with the morning cycle and performed simultaneously for all experimental groups.

### Immunofluorescence

Flies were frozen in dry ice immediately after sleep deprivation. Brains were dissected and fixed with 4% formaldehyde in 100 mm phosphate buffer (PBS) for 30 min at room temperature. The brains were then washed and rinsed four times in PBS with 0.3% Triton X-100 (PB-T) for 10 min to remove the formaldehyde. Brains were then were blocked in 5% normal goat serum in PB-T for 1 h and incubated with primary antibody (rabbit anti-RFP 1:1000; Invitrogen), at 4°C overnight in a humid chamber. The next day, the brains were washed four times for 10 min each time in PB-T and incubated with secondary antibody at 1:500 for 2 h at RT. The secondary antibody was washed four times for 10 min each time in PB-T. To remove the PB-T, the brains were washed two times for 5 min with PBS and mounted in 80% Prolong Anti-fading Agent. Images were taken either on a Zeiss Pascal LSM or a Zeiss LSM 510 Meta confocal microscope at 40X magnification. The same gain was used for all images after calibrating the gain with the group of brains of the *pum*^RNAi^ deprived flies, which showed the highest level of fluorescence of all experimental groups. After acquisition, images were processed employing Imaris (Redicon) software program.

### Statistical Methods

All statistical comparisons for significance between control and experimental groups was calculated using a significance cut off *p* < 0.05. All statistical analyses were performed using GraphPad Prism 8 software. Statistical analyses performed are included in the figure legends.

## Data Availability Statement

The raw data supporting the conclusions of this article will be made available by the authors, without undue reservation, to any qualified researcher.

## Author Contributions

JA, NR, and LD designed the study. JA-R, NR, CP-A, JO-T, RN, MF, and LD performed the experiments and data analysis. JA, AG, NF-U, and LD wrote and reviewed the manuscript.

## Conflict of Interest

The authors declare that the research was conducted in the absence of any commercial or financial relationships that could be construed as a potential conflict of interest.
