# Supplementary material for: Pumilio Regulates Sleep Homeostasis in Response to Chronic Sleep Deprivation in Drosophila melanogaster
Source: Front Neurosci. 2020 Apr 17;14:319. doi: 10.3389/fnins.2020.00319 (PMC7182066; doi:10.3389/fnins.2020.00319)
Supplement: Supplementary file 1 [file Data_Sheet_1.pdf]

pumilio regulates sleep homeostasis in response to chronic sleep deprivation in *Drosophila melanogaster*

Luis A. De Jesús-Olmo[1], Norma Rodríguez[1], Marcelo Francia[1], Jonathan Alemán-Rios[1], Carlos J. Pacheco-Agosto[1], Joselyn Ortega-Torres[1], Richard Nieves[1], Nicolás Fuenzalida-Uribe[1], Alfredo Ghezzi[1], José L. Agosto[1]*

[1]Department of Biology, University of Puerto Rico, Rio Piedras, P.R., USA.

Supplementary Information

Supplementary Results

Knockdown of *pumilio* affects gene expression profile after sleep deprivation

To determine if the reduction in homeostatic sleep rebound observed between acute vs chronic SD *pum*^{RNAi} flies can be explained by differential changes in gene expression, we performed a quantitative reverse-transcription polymerase chain reaction (qRT-PCR) for a selected group of genes encoding synaptic proteins, synaptic translation modulators, neurotransmitter receptors and ion channels. For our analysis, we selected the synaptic genes *bruchpilot* (*brp*), *disks large 1* (*dlg1*) and *Synapsin* (*Syn*) as their protein products are known to increase after acute SD, as shown by western blots of whole fly brains (Gilestro, et al., 2009). In addition, we selected three genes that encode translation regulators —the *eukaryotic translation initiation factor 4E1* (*eIF4E1*), *Target of rapamycin* (*Tor*), and the Protein Kinase B (*Akt1*) because, as previously stated, EIF4E is a direct Pum target and both TOR and AKT are upstream regulators of EIF4E (Miron, et al., 2003). We also included genes for the voltage gated sodium channel *paralytic (para)*, the voltage gated potassium channel *Shaker cognate l* (*Shal*) and *slowpoke* (*slo*), and the potassium channel modulator *sleepless* (*sss,* also known as *quiver* (*qvr*)), due to their relation to neuronal excitability. To complete the qRT-PCR testing panel, we also included the nicotinic Acetylcholine Receptor gene (*nAchRα1*), the $GABA_A$ receptor gene *Resistant to dieldrin* (*Rdl*) and the *Glutamic acid decarboxylase 1* (*Gad1*) gene, which encodes for the enzyme that synthesizes the inhibitory neurotransmitter GABA (Lee, et al., 2003), because they also have been associated to regulations in neuronal excitability (see supplementary table S1 for references).

The RNA for the qRT-PCR study was extracted from whole heads, which were frozen two hours after the completion of the SD protocol. We evaluated the gene expression for non-deprived conditions against acute SD (12 hours) and chronic SD (84 hrs). The non-deprived results come from flies of each of the phenotypes handled in parallel to the deprived flies during the same experimental dates. Pum expression levels using qRT-PCR of whole heads showed a 50% expression reduction at baseline in *pum*^{RNAi} non-deprived flies (Fig. S1), which is expected for an RNAi knockdown. Surprisingly, the same flies showed an increase in expression after SD (Fig. S1).

We assessed the effects of *pum* knockdown within non-deprived flies on basal gene expression of our selected gene panel. The results show a significantly increased expression of *Shal* and *Gad1* in *pum*^{RNAi} flies as compared to the "sibling" controls (Supplementary Fig. S5). The expression increase in the inhibitory neurotransmitter synthesis enzyme *Gad1* was expected because Gad1 is a predicted target of Pum (Chen, et al., 2008). Furthermore, it has been shown that GABA acts as a slow inhibitory neurotransmitter in circadian neurons (Hamasaka, et al., 2005), promoting fly sleep (Parisky, et al., 2008). The fact that *pum*^{RNAi} flies showed increase levels of *Shal* and *Gad1* in non-deprived flies, suggests that the presence of Pum is also necessary to maintain normal sleep. This fact was corroborated by the increase in baseline sleep of *pum*^{RNAi} flies (supplementary Fig. S2), which should be expected under increased GABAergic inhibition of wake promoting neurons (Parisky, et al., 2008).

Next, we assessed the changes in gene expression induced by acute and chronic SD, in both *pum*^{RNAi} flies and "sibling" controls. The qRT-PCR results showed that four genes displayed significant expression changes after acute SD but no change in response to chronic SD. These genes are: *nAchRα1*, *Rdl*, *para* and *slo* (Fig. S5B-E). For *nAchRα1*, this change was exacerbated in the *pum*^{RNAi} flies, whereas for *Rdl*, *para* and *slo*, the effect of acute SD in expression observed in control flies was abolished by the knockdown in *pum*. In contrast, eight different genes displayed significant changes between *pum*^{RNAi} flies and "sibling" controls in response to chronic SD, but no change in response to acute SD (Fig. S5F-M). A *pum* knockdown-dependent increase was observed in *eIF4E1, Tor, Akt, brp, dlg,* and *Shal,* whereas a *pum* knockdown-dependent decrease was observed in *Syn* and *Gad1*. These results showed a concordance between the selected markers overexpressed by *pum's* knockdown and their association with increased neuronal excitability (see supplementary Table 1for references). In addition, the combined results confirmed our hypothesis that acute vs chronic SD exhibit differential gene expression patterns, which points towards a differential regulation of acute vs chronic SD.

Supplementary Materials and Methods

Measurement of gene expression by qRT-PCR: RNA was extracted from heads of adult flies using the Qiagen RNeasy Mini kit (Qiagen, Crawley, UK). Five heads were pooled to make one sample and homogenized with a plastic mortar in 100ul of lysis buffer containing 0.1 M-mercaptoethanol, then 250 ul of lysis buffer was added and centrifuged. 350 ul of 70% ethanol was added and passed through a RNeasy column. After washing in buffer, immobilized nucleic acids were then treated with 190 U of DNase I for 15 min, washed again in stages according to manufacturer's protocol, and then eluted in 20 ul of RNase-free water. Quantification of RNA concentration was made using a ND-1000 Nanodrop spectrophotometer (Nanodrop, Wilmington, DE). All extracted RNA samples were analyzed to assure quality using the Agilent Bioanalyzer, any samples showing signs of degradation were discarded. After adjusting for concentration, synthesis of cDNA was performed with the iScript Reverse transcription Supermix (Bio-Rad) as per manufacturer protocol. The mix was incubated at 25 ^{o}C for 5 min, then at 42 ^{o}C for 30 min followed by 85°C for 5 min to inactivate reverse transcription. From the total reaction volume of 20ul, 1 ul of cDNA was used for each PCR sample. All primers were obtained from Integrated DNA Technologies. An Eppendorf Mastercycler Thermal Cycler was used for the relative quantification of target mRNAs. Reactions contained 5 ul of Syber green (SYBR) (Invitrogen), 0.5 ul of each forward and reverse primer (both 10 mM), 3 ul of water, and 1 ul of cDNA. Cycling was as follows: initial denaturation of 15 sec at 95°C, then 40 cycles of annealing for 60 sec. for all primer pairs used, extension at 65°C for 1:20 min and melting curve generation at 95°C. Each group of 7 samples were tested in triplicate. Final mRNA levels were expressed as relative fold change normalized

against *rp49* mRNA. The comparative cycle threshold (Ct) method (User Bulletin 2, 1997; Applied Biosystems, Foster City, CA) was used to analyze the data.

Supplementary Figures

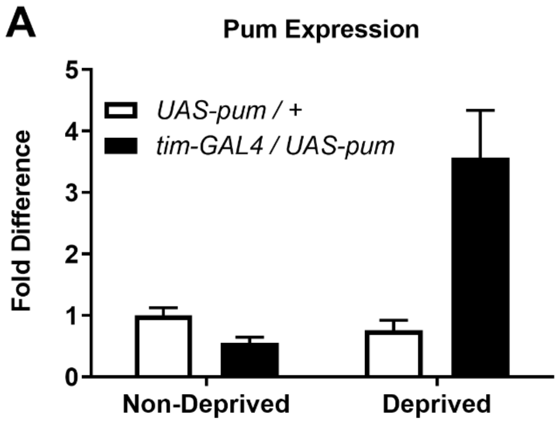

Figure S1: Pum^{RNAi} knockdown reduces pum expression level during baseline sleep. (A) *pum* expression at baseline and after chronic SD comparing *UAS-pum*^{RNAi}/ + sibling control fies vs *UAS-pum*^{RNAi}/ *tim-GAL4* experimental flies. A Two-way ANOVA showed a significant difference in the factors Interaction (F (1, 71) = 14.37; P=0.0003) Deprivation (F (1, 71) = 10.44; P=0.0019) and Genotype (F (1, 71) = 7.547; P=0.0076). Data points and error bars represent means ± SEM. Stars indicate significance level (* denotes p<0.05; ** p< 0.01; *** p< 0.001; **** p< 0.0001).

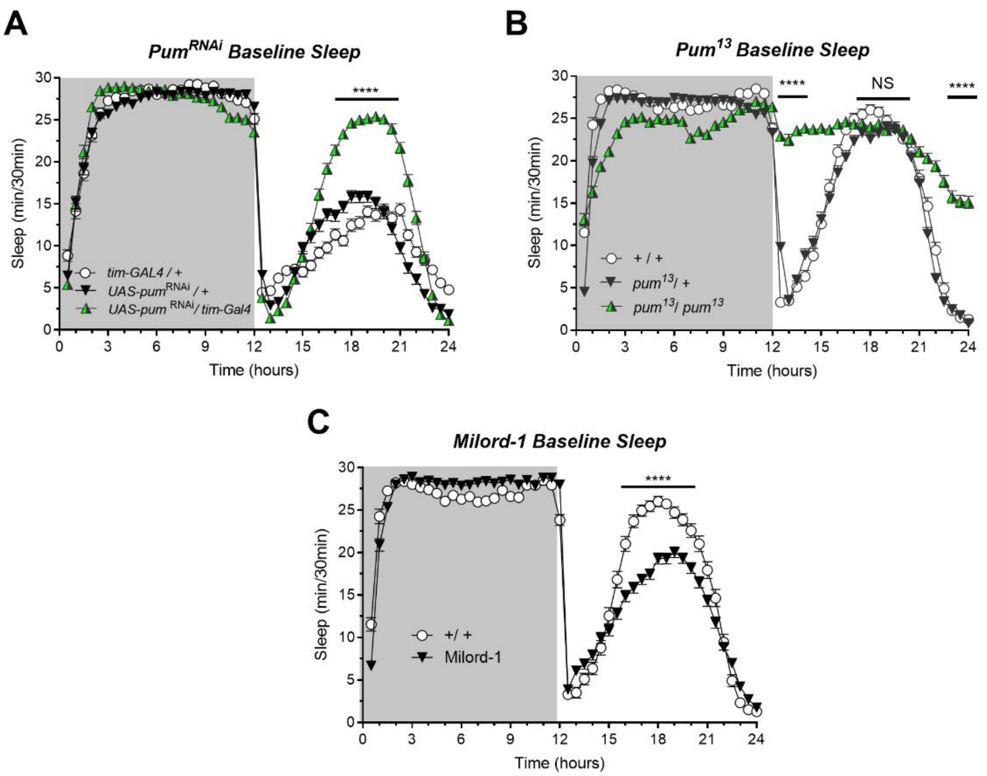

Figure S2: Transgenic flies showed increase baseline sleep. (A) Graphs showing the average sleep activity for UAS-*pum*^{RNAi}/+ ("sibling" control) and UAS-*pum*^{RNAi}/*tim*-Gal4 under baseline sleep conditions compared to parental *tim*-Gal4/+ baseline. The y-axis shows the number of minutes that flies slept in intervals of 30 min. **(B)** Graph showing baseline sleep for all *pum*^{13} lines. **(C)** Graph showing baseline sleep for Milord-1 line. Stars indicate significance level (* denotes $p<0.05$; ** $p< 0.01$; *** $p< 0.001$; **** $p< 0.0001$).

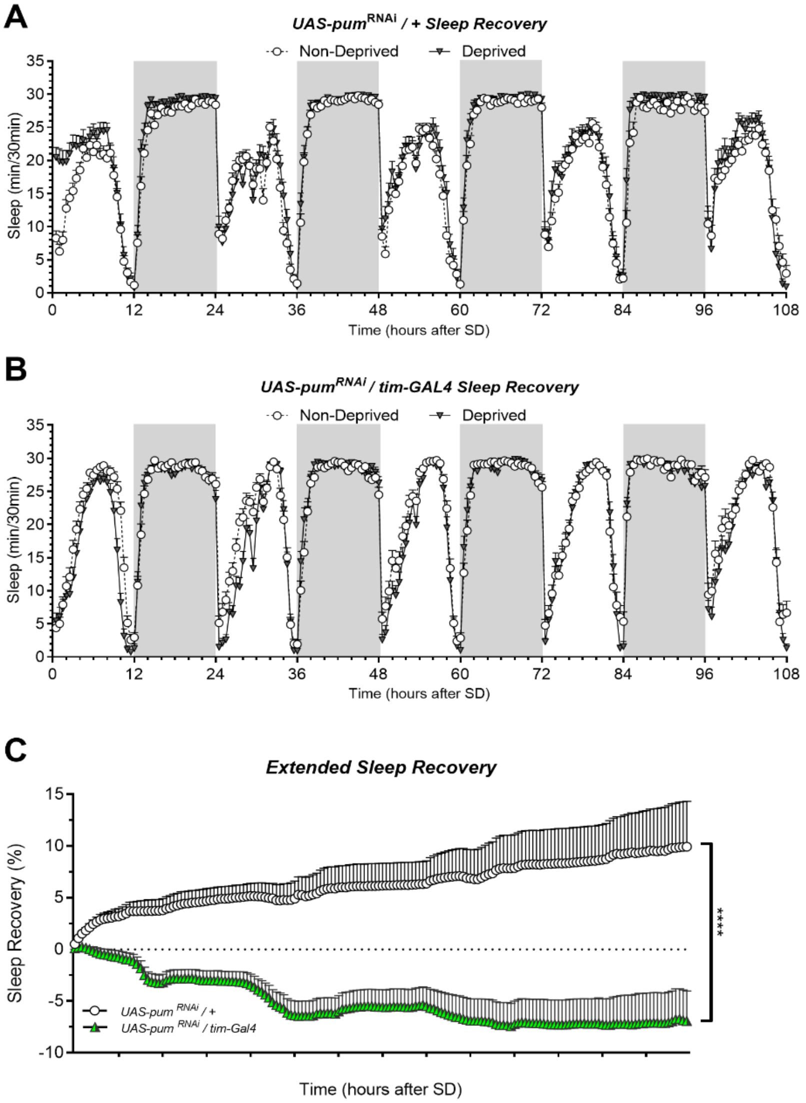

Figure S3: *Pum* knockdown shows reduced sleep recovery up to 96 hours after chronic sleep deprivation. Sleep comparison of UAS-*pum*^{RNAi}/*tim*-Gal4 (experimental) vs UAS-*pum*^{RNAi}/+ ("sibling" controls) for the period following chronic SD. **(A-B)** Depiction of sleep activity during the recovery period for both genotypes after chronic mechanical SD. **(C)** Extended percent sleep recovery after SD. Graph depicting up to 108 hours of sleep recovery after chronic SD. Two-Way ANOVA with repeat measures indicated significant differences between the genotypes (F (1, 80) = 18.1 P<0.0001) and interaction (F (167, 13360) = 8.362 P<0.0001). Post-hoc analysis using Tukey's multiple comparisons test revealed significant differences between UAS-*pum*^{RNAi}/*tim*-Gal4 throughout the recovery period. The y-axis shows the number of minutes that flies slept in intervals of 30min. The data shown represents two experiments with the following sample sizes (N): UAS-*pum*^{RNAi}/+ Non-Deprived (N=60) and Deprived (N=39); UAS-*pum*^{RNAi}/*tim*-Gal4 Non-Deprived (N=63) and Deprived (N=43). Because the calculations of sleep lost and sleep recovery involve both the Non-Deprived and Deprived groups (see methods), the N for panels A and B is equal to the N of the Deprived group. The y-axis shows the number of minutes that flies slept in intervals of 30min. Stars indicate significance level (* denotes p<0.05; ** p< 0.01; *** p< 0.001; **** p< 0.0001.

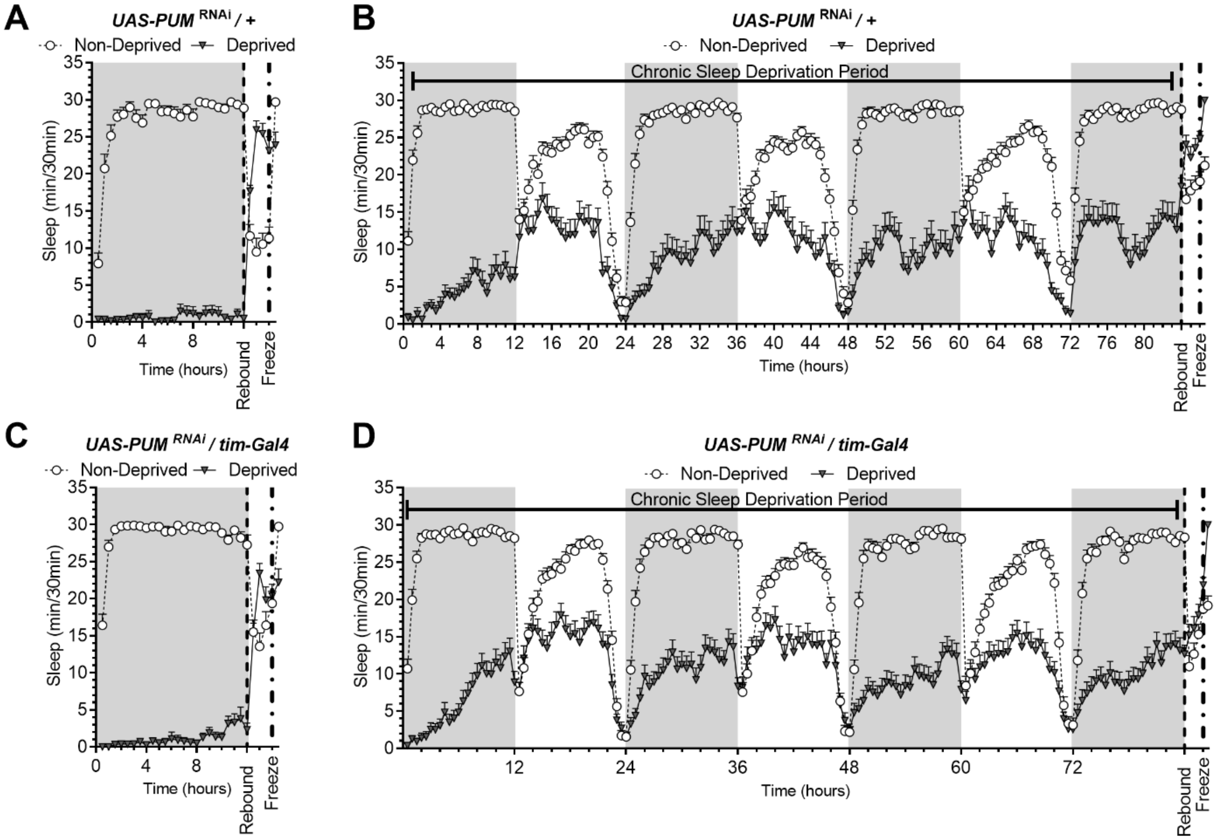

Figure S4: *Pum*^{RNAi} acute SD time course from qRT-PCR flies confirmed acute SD differences in sleep rebound Sleep comparison of *UAS-pum*^{RNAi}/*tim*-Gal4 (experimental) vs UAS-*pum*^{RNAi}/+ ("sibling" controls) during acute and chronic SD. Flies were removed from the monitors after two hours of sleep recovery and immediately freeze for qRT-PCR analysis. **(A,C)** Depiction of the acute sleep deprivation and sleep rebound period for both genotypes. The y-axis shows the number of minutes that flies slept in intervals of 30 min. The data shown represents one experiment with the following sample sizes (N): UAS-*pum*^{RNAi}/+ Non-Deprived (N=31) and Deprived (N=27); UAS-*pum*^{RNAi}/*tim*-Gal4 Non-Deprived (N=31) and Deprived (N=32). **(B,D)** Depiction of the sleep deprivation period and sleep rebound pattern for *tim*-Gal4/+ (parental) flies, UAS-*pum*^{RNAi}/+ ("sibling") and UAS-*pum*^{RNAi}/*tim*-Gal4 exposed to chronic (84hrs) mechanical SD. The data shown represents two experiments with the following sample sizes (N): UAS-*pum*^{RNAi}/+ Non-Deprived (N=62) and Deprived (N=34); UAS-*pum*^{RNAi}/*tim*-Gal4 Non-Deprived (N=61) and Deprived (N=54). Error bars represent means ± SEM.

Pumilio regulates sleep homeostasis in response to chronic sleep deprivation in *Drosophila melanogaster*

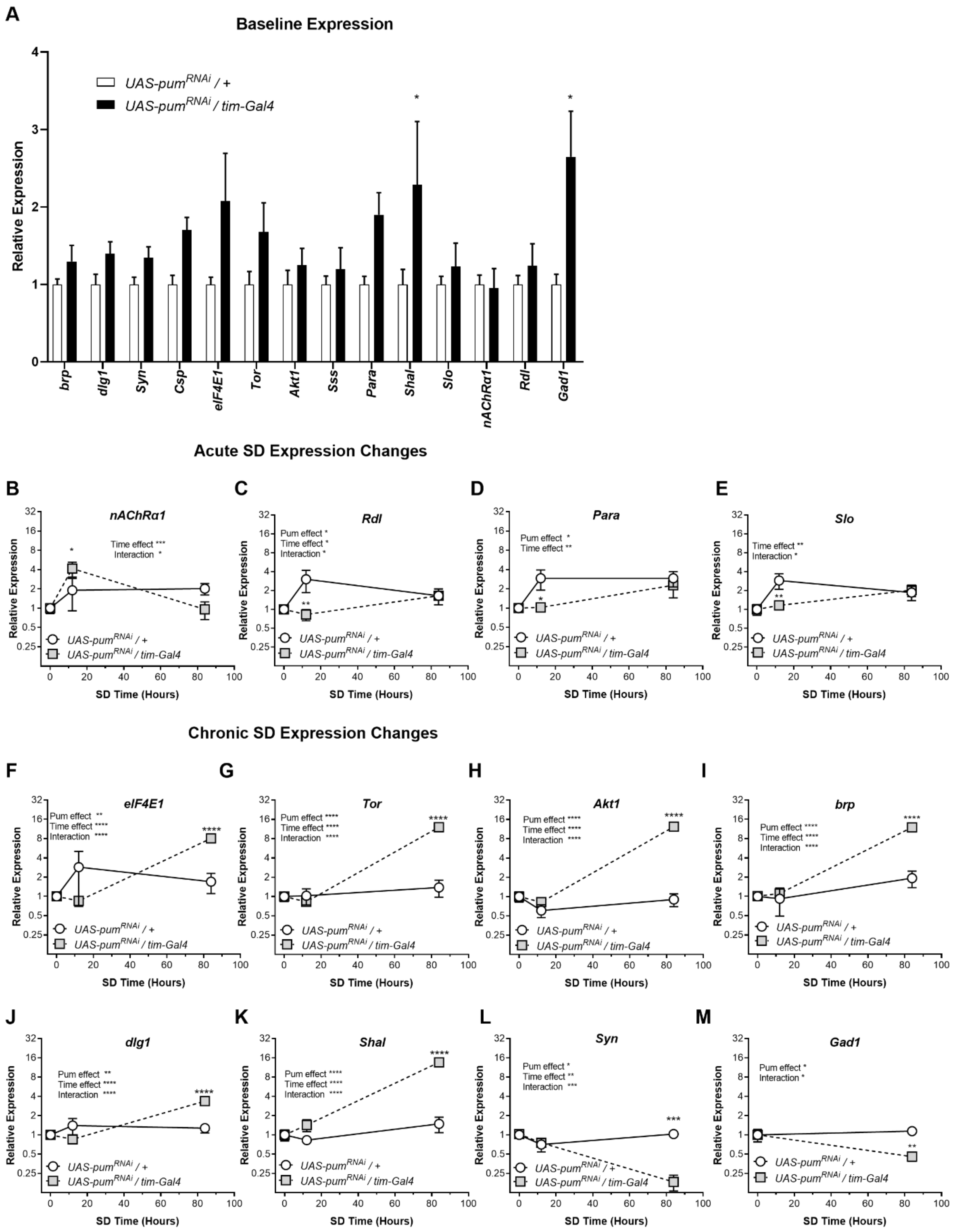

Figure S5: Pum knockdown results in differential gene expression patterns between acute (12 hours) and chronic (84 hours) sleep deprivation. Gene expression comparison of UAS-*pum*^{RNAi}/+ ("sibling" controls) vs UAS-*pum*^{RNAi}/*tim*-Gal4 (experimental) subjected to acute (12 hours) mechanical SD vs chronic SD. **(A)** Baseline gene expression in non-deprived flies from both genotypes. **(B-E)** Time-course plots for non-deprived, acutely deprived and chronically deprived flies showing expression changes during acute deprivation. The fold change is expressed in log scale. **(F-J)** Time-course plots for non-deprived, acutely deprived and chronically deprived flies showing expression changes during chronic SD. The fold change is expressed in log scale. Data points and error bars represent means ± SEM. Two-way Analysis of variance (ANOVA) with repeated measures revealed significant effects due to *pum*, Time (T=0, T=12, T=84 hrs SD) and interactions between the parameters in some of the groups (see graphs for results). Stars indicate significance level (* denotes $p<0.05$; ** $p< 0.01$; *** $p< 0.001$; **** $p< 0.0001$).

Supplementary Table

Table S1: Summary of PR-PCR results in relation to each marker's effect in neuronal excitability.

Gene	Gene Name	Results	Description	Relation to neuronal activity	References
brp	*bruchpilot*	Over-expressed due to *pum* effect	Scaffolding synaptic protein	Increase amplitude of miniature excitatory junctional currents (mEJCs)	Kittel, et al., 2006
dlg1	*discs large 1* (PSD-95)	Over-expressed due to *pum* effect	Scaffolding synaptic protein	Enhances excitatory synapse size and miniature frequency	Prange, et al., 2004
Syn	*Synapsin*	Under-expressed due to *pum* effect	Synaptic protein	Knockdown increases spontaneous and evoked activities	Chiappalone, et al., 2009
Csp	*Cysteine string protein*	No change	Synaptic protein	Promotes neuronal homeostasis	Brusich, et al., 2015
eIF4E1	*eukaryotic translation initiation factor 4E1*	Over-expressed due to *pum* effect	Translational regulation	Promotes retrograde compensatory enhancement in neurotransmitter release	Penney, et al., 2012
Tor	*Drosophila Target of rapamycin*	Over-expressed due to *pum* effect	Translational regulation	Promotes retrograde compensatory enhancement in neurotransmitter release	Penney, et al., 2012
Akt1	*Akt1* (Protein Kinase B)	Over-expressed due to *pum* effect	Serine/Threonine Kinase	Knockdown prevented the insulin-induced increase in dendritic spine density	Lee, et al., 2011
sss	*quiver* (sleepless)	No change	Ion channel regulator	Decreases neuronal excitability by antagonizing nicotinic acetylcholine receptors (nAChRs)	Wu, et al., 2014
Para	*paralytic*	No change in chronic SD<br>Under-expressed in Acute SD *pum*^{RNAi}	Ion channel (Na(v))	Increase neuronal excitability	Mee, et al., 2004

Shal	*Shaker cognate l*	Over-expressed due to *pum* effect	Ion channel (K)	Increases neuronal excitability when mutated, reduces excitability when open.	Parrish, et al., 2014<br><br>Ottosson, et al., 2015
Slo	*slowpoke*	No change in chronic SD<br>Under-expressed in Acute SD in *pum*^{RNAi}	Ion channel (BK)	Increases neuronal excitability by shortening refractory period<br>Effects in neuronal excitability are circuit dependent	Ghezzi & Atkinson, 2011<br><br>Jepson, et al., 2013
nAChRα1	*nicotinic Acetylcholine Receptor*	No change	Ion channel	Increases neuronal excitability	Wu, et al., 2014
Rdl	*resistant to dieldrin*	No change in chronic SD<br>Under-expressed Acute SD in *pum*^{RNAi}	Ion channel ($GABA_A$)	Decreases neuronal excitability	Parisky, et al., 2008
Gad1	*Glutamic acid decarboxylase 1*	Over-expressed due to *pum* effect<br>Under-expressed after Chronic SD due to *pum* effect	$GABA_A$ synthetase	Chemical increase in GABA rescued larvae from lethality associated with reduced excitability	Li, et al., 2014

References:

Brusich, DJ., Spring, AM., Frank, CA. A single-cross, RNA interference-based genetic tool for examining the long-term maintenance of homeostatic plasticity. Front Cell Neurosci. Mar 26;9:107.

Chiappalone, M., Casagrande, S., Tedesco, M., Valtorta, F., Baldelli, P., Martinoia, S., et al. (2009). Opposite changes in glutamatergic and GABAergic transmission underlie the diffuse hyperexcitability of synapsin I-deficient cortical networks. Cereb Cortex. 19(6),1422-39.

Chen, G., Li, W., Zhang, Q. S., Regulski, M., Sinha, N., Barditch, J., Tully, T., Krainer, A. R., et al. (2008). Identification of synaptic targets of Drosophila pumilio. PLoS computational biology, 4(2).

Ghezzi, A., Atkinson, NS. (2011). Homeostatic control of neural activity: a Drosophila model for drug tolerance and dependence. Int Rev Neurobiol. 99, 23-50.

Gilestro, GF., Tononi, G., Cirelli, C. 2009. Widespread changes in synaptic markers as a function of sleep and wakefulness in Drosophila. Science. Apr 3;324(5923):109-12.

Hamasaka, Y; Wegener, C; Nasser, DR. (2005). GABA modulates Drosophila circadian clock neurons via GABAB receptors and decreases in calcium. Journal of Neurobiology, 65(3):225-240.

Jepson, J., Sheldon, A., Shahidullah, M., Fei, H., Koh, K., Levitan, IB. (2013) Cell-specific fine-tuning of neuronal excitability by differential expression of modulator protein isoforms. J Neurosci. Oct 16;33(42):16767-77.

Kittel, RJ., Wichmann, C., Rasse, TM., Fouquet, W., Schmidt, M., Schmid, A., et al. (2006). Bruchpilot promotes active zone assembly, Ca2+ channel clustering, and vesicle release. Science. 312(5776),1051-4.

Lee, CC., Huang, CC., Hsu, KS. (2011). Insulin promotes dendritic spine and synapse formation by the PI3K/Akt/mTOR and Rac1 signaling pathways. Neuropharmacology. 61(4), 867-79.

Lee, D., Su, H., O'Dowd DK. (2003). GABA receptors containing Rdl subunits mediate fast inhibitory synaptic transmission in Drosophila neurons. J Neurosci. 23(11), 4625– 4634.

Li, X., Overton, IM., Baines, RA., Keegan, LP., O'Connell, MA. (2014). The ADAR RNA editing enzyme controls neuronal excitability in Drosophila melanogaster. Nucleic Acids Res. 42(2):1139-51.

Miron, M., Lasko, P., Sonenberg N. (2003). Signaling from Akt to FRAP/TOR targets both 4E-BP and S6K in Drosophila melanogaster. Mol Cell Biol. Dec;23(24):9117-26.

Parisky, KM., Agosto, J., Pulver, SR., Shang, Y., Kuklin, E., Hodge, J., et al. (2008). PDF Cells Are a GABA-Responsive Wake-Promoting Component of the Drosophila Sleep Circuit. Neuron 61(1).

Penney, J., Tsurudome, K., Liao, EH., Elazzouzi, F., Livingstone, M., Gonzalez, M., et al. (2012). TOR is required for the retrograde regulation of synaptic homeostasis at the Drosophila neuromuscular junction. Neuron. 74(1),166-78.

Prange, O., Wong, TP., Gerrow, K., Wang, YT., El-Husseini, A. (2004). A balance between excitatory and inhibitory synapses is controlled by PSD-95 and neuroligin. Proc Natl Acad Sci U S A. 101(38),13915-20.

Wu, M., Robinson, JE., Joiner, WJ. (2014). SLEEPLESS is a bifunctional regulator of excitability and cholinergic synaptic transmission. Curr Biol. 24(6), 621-9.
